# Right Ventricular Damage in COVID-19: Association Between Myocardial Injury and COVID-19

**DOI:** 10.3389/fcvm.2021.606318

**Published:** 2021-02-16

**Authors:** Yonghao Lan, Wei Liu, Yujie Zhou

**Affiliations:** ^1^Department of Cardiology, Beijing Jishuitan Hospital, Peking University Fourth Hospital, Beijing, China; ^2^Department of Cardiology, Beijing Anzhen Hospital, Capital Medical University, Beijing, China; ^3^Beijing Key Laboratory of Precision Medicine of Coronary Atherosclerotic Disease, Clinical Center for Coronary Heart Disease, Beijing Institute of Heart Lung and Blood Vessel Disease, Capital Medical University, Beijing, China

**Keywords:** COVID-19, right ventricular damage, myocardial injury, cardiovascular magnetic resonance, echocardiography, ARDS

## Abstract

Coronavirus disease 2019 (COVID-19), caused by severe acute respiratory syndrome coronavirus 2, is a global pandemic. It has resulted in considerable morbidity and mortality around the world. The respiratory system is the main system invaded by the virus involved in COVID-19. In addition to typical respiratory manifestations, a certain proportion of severe COVID-19 cases present with evidence of myocardial injury, which is associated with excessive mortality. With availability of an increasing amount of imaging data, right ventricular (RV) damage is prevalent in patients with COVID-19 and myocardial injury, while left ventricular damage is relatively rare and lacks specificity. The mechanisms of RV damage may be due to increased RV afterload and decreased RV contractility caused by various factors, such as acute respiratory distress syndrome, pulmonary thrombosis, direct viral injury, hypoxia, inflammatory response and autoimmune injury. RV dysfunction usually indicates a poor clinical outcome in patients with COVID-19. Timely and effective treatment is of vital importance to save patients' lives as well as improve prognosis. By use of echocardiography or cardiovascular magnetic resonance, doctors can find RV dilatation and dysfunction early. By illustrating the phenomenon of RV damage and its potential pathophysiological mechanisms, we will guide doctors to give timely medical treatments (e.g., anticoagulants, diuretics, cardiotonic), and device-assisted therapy (e.g., mechanical ventilation, extracorporeal membrane oxygenation) when necessary for these patients. In the paper, we examined the latest relevant studies to investigate the imaging features, potential mechanisms, and treatments of myocardial damage caused by COVID-19. RV damage may be an association between myocardial damage and lung injury in COVID-19. Early assessment of RV geometry and function will be helpful in aetiological determination and adjustment of treatment options.

## Introduction

Coronavirus disease 2019 (COVID-19) has spread rapidly and triggered a terrible global pandemic that involves more than 200 countries/regions. On 6 December 2020, there were more than 66.9 million confirmed cases and 1,534,954 deaths internationally (1). Although respiratory symptoms are usually predominant in COVID-19, elevated troponin levels have been found at the early stage in some cases, indicating that COVID-19 also affects the heart. In particular, there is an increased prevalence of cardiovascular complications, including new or worsening heart failure, arrhythmia, acute myocarditis, and myocardial infarction, in severe and critically ill patients with COVID-19. Recent studies have shown that the incidence of acute myocardial injury in hospitalized patients with COVID-19 is ~20–28% (2–4). With an increase in imaging evidence, such as echocardiography and magnetic resonance imaging (MRI), right ventricular (RV) involvement has been observed more commonly than left ventricular (LV) involvement in patients with COVID-19, with ~40% of patients experiencing RV dilatation and RV dysfunction (5, 6). RV damage is associated with a higher incidence of myocardial damage in COVID-19 and generally predicts a worse prognosis (7). This review aims to describe involvement of RV damage in patients with COVID-19, to determine the association of RV damage with COVID-19 and its plausible mechanisms, and to summarize the existing appropriate treatment strategies to improve patients' prognosis.

## Myocardial Injury in Covid-19 is Common

Previous influenza-related studies have shown that elevated cardiac enzymes are relatively uncommon (8). Cardiac abnormalities associated with influenza are usually subclinical and/or transient (9). However, COVID-9-related cardiac injury is significantly different from influenza. In a review of 26 studies that included 11,685 patients, the overall prevalence of COVID-19-related acute myocardial injury ranged from 5 to 38% (10). N-terminal pro-brain natriuretic peptide and cardiac troponin-I levels were shown to be significantly higher in critically ill patients with COVID-19 than in non-critically ill patients (2). These findings suggest that the magnitude of elevated cardiac troponin levels may be related to the severity and prognosis of the disease (11). Monitoring cardiac troponin-I levels is important for judging the status of COVID-19, while understanding myocardial injury in patients with COVID-19. Chinese guidelines recommend myocardial enzyme monitoring in patients who are admitted for COVID-19 (12). Troponins are often associated with LV ischaemia and infarction. However, previous studies have shown that the most common mechanism of elevated troponin levels in patients with COVID-19 is acute RV damage rather than LV functional impairment (5). Specific manifestations of myocardial structural damage require assessment of cardiac imaging. Early retrospective analysis did not show any specificity between electrocardiography and echocardiography (13). However, with publication of more imaging study results, there are particularities in cardiac structural changes. Therefore, imaging assessment of cardiac injury in COVID-19 is important and helpful for differential diagnosis of cardiac events.

## RV Involvement From Cardiac Images in Patients With Covid-19

With the discovery of COVID-19-related myocardial damage, cardiac imaging is becoming more common, and it can help to better understand the structural characteristics of COVID-19-related myocardial damage. Imaging studies can not only detect lesions, but also guide further treatment. We searched PubMed, EMBASE, and Web of Science until August 2020 for RV clinical research. “Snowball sampling” by searching reference lists and citation tracking was performed in each retrieved article. No language restrictions were applied. Following search terms were used: (“magnetic resonance imaging” OR “echocardiography” OR “myocardial injury” OR “cardiac manifestations” OR “cardiac function” OR “right ventricular damage/injury” OR “right ventricular dysfunction” OR “right ventricular dilatation”) AND (“coronavirus” OR “SARS-COV-2” OR “COVID-19”). Recent findings on imaging assessment of cardiac injury in COVID-19 were summarized in Tables 1, 2.

**Table 1:** Studies of CMR imaging assessments in patients with COVID-19 and cardiac injury.

**Study, publish date**	**Study type**	**Location**	**Study period**	**Patients**	**Image type**	**Mean age & gender**	**Main test items**	**Main findings**
Huang et al., May 4, 2020 (14)	Retrospective study	Tongji Hospital, Tongji Medical College, Wuhan, China	Since March, 2020	26 hospitalized patients, recovered from COVID-19 with cardiac symptoms, no previous cardiac disease or COPD	CMR	32–45 26% male	♢ Conventional sequences (cine, T2WI, LGE) ♢ Quantitative mapping sequences (T1, T2, T1/T2, ECV mapping) ♢ Oedema ratio ♢ Cardiac function	♢ 15 (58%) T2 signal ↑ and/or positive LGE ♢ 14 (54%) myocardial oedema ♢ Global native T1, T2, ECV values ↑ in COVID-19 patients with positive cardiac MRI findings ♢ RVEF, CO, CI, SV, SV/BSA ↓ in COVID-19 patients with positive cardiac MRI findings ♢ No significant differences of LV function among controls and patients
Puntmann et al., July 27, 2020 (15)	Prospective observational cohort study	University Hospital Frankfurt COVID-19 Registry, Germany	April to June, 2020	100, recovered from COVID-19 including mostly home-based recovery and hospitalized patients, 13% prior CAD, 21% prior COPD or asthma	CMR	45–53 53% male	♢ LVEF ♢ LVEDV index ♢ LV mass index ♢ RVEF ♢ Native T1 and T2 ♢ LGE ♢ Pericardial effusion	♢ LVEF ↓ ♢ RVEF ↓ ♢ 78% abnormal CMR: 73% native T1 ↑, 60% native T2 ↑, 32% myocardial LGE, 22% pericardial LGE ♢ LV volume and mass ↑ ♢ High-sensitivity troponin T was significantly correlated with native T1, native T2 and LV mass ♢ Native T1 and T2 were the best measures to detect COVID-19-related myocardial pathology
Rajpal et al., Sep 11, 2020 (16)	Prospective study	Ohio State, USA	June 2020 to August 2020	26 competitive college athletes, recovered from COVID-19 without hospitalization, no previous cardiac disease or COPD	CMR	19.5 ± 1.5 57.7% male	♢ LGE ♢ LVEF and RVEF ♢ T1 and T2 mapping ♢ LVEDV and RVEDV	♢ 4 athletes had CMR findings consistent with myocarditis ♢ 12 (46%) had LGE, of whom 8 (30.8%) had LGE without concomitant T2 elevation ♢ Mean (SD) T2 in those with suspected myocarditis was 59 ms compared with 51 ms in those without myocarditis. ♢ CMR may provide an excellent riskstratification assessment for myocarditis in athletes who have recovered from COVID-19.

*BSA, body surface area; CI, cardiac index; CMR, cardiovascular magnetic resonance; CO, cardiac output; COVID-19, coronavirus disease 2019; ECV, extracellular volume; LV, left ventricular; LVEDV, left ventricular end-diastolic volume; LVEF, left ventricular ejection fraction; LGE, late gadolinium enhancement; RVEF, right ventricular ejection fraction; SV, stroke volume; T2WI, T2-weighted imaging*.

**Table 2:** Studies of echocardiography assessments in patients with COVID-19 and cardiac injury.

**Study, publish date**	**Study type**	**Location**	**Study period**	**Patients**	**Image type**	**Mean age & gender**	**Main test items**	**Main findings**
Argulian et al., May 7, 2020 (17)	Retrospective study	Mount Sinai Morningside Hospital, New York, USA	March 26, 2020 to April 22, 2020	105 hospitalized patients, 31 of whom were intubated and mechanically ventilated during examination	TTE	66 ± 14.6 64% male	♢ RV and LV sizes and function	♢ 32 (31%) RV dilatation ♢ Renal dysfunction is more common in patients with RVD than those without ♢ No differences in LV size and function ♢ 21 (20%) patients died: 13 (41%) deaths were observed in patients with RV dilatation and 8 (11%) in patients without RV dilatation ♢ RV enlargement was significantly associated with mortality
Li et al., April 24, 2020 (7)	Retrospective study	The west branch of Union Hospital, Tongji Medical College, Wuhan, China	February 12, 2020 to March 15, 2020	120 hospitalized patients (Survivors 102 and non-survivors 18), 9.2% prior CVD, 5% prior COPD	TTE, examed in 3–10 days	61 ± 14 58% male	♢ RVFAC ♢ TAPSE ♢ Tricuspid tissue Doppler annular velocities (S') ♢ RVLS ♢ LV volume and function	♢ Male, ARDS, RVLS, RVFAC and TAPSE were significant univariate predictors of higher risk for mortality ♢ RVLS was found to predict higher mortality more accurately ♢ The best cut-off value of RVLS for prediction of outcome was −23%
Szekely et al., May 29, 2020 (5)	Prospective study	Tel Aviv Medical Center, Israel	March 21, 2020 to April 16, 2020	100 hospitalized patients, 16% prior IHD	TTE, examed within 24 h	66.1 ± 17.3 63% male	♢ LV systolic and diastolic function ♢ Valve hemodynamics ♢ RV assessment (TAPSE, RV-S', RVFAC, Tei index, pulmonary acceleration time) ♢ Lung ultrasound	♢ 32% normal echocardiography ♢ 39% RV dilatation with or without dysfunction ♢ 16% LV diastolic dysfunction ♢ 10% LV systolic dysfunction ♢ Patients with elevated troponin (20%) or worse clinical condition had worse RV function
Mahmoud-Elsayed et al., May 24, 2020 (6)	Retrospective study	Queen Elizabeth Hospital Birmingham, United Kingdom	March 22, 2020 to April 17, 2020	74 hospitalized patients, referred for TTE with ≥ 1 clinical indication(s), 9% prior CAD	TTE, examed in 3–10 days	59 ± 13 78% male	♢ Chamber sizes and function ♢ Valvular disease ♢ Pulmonary hypertension	♢ 41% RV dilatation ♢ 27% RVD ♢ 89% LV function was hyper-dynamic or normal ♢ RV impairment was associated with increased D-dimer and CRP levels
Jain et al., June 9, 2020 (18)	Retrospective study	Columbia University Irving Medical Center and New York-Presbyterian Allen Hospital, New York, USA	March 1, 2020 to April 3, 2020	72 hospitalized patients, referred for TTE when having clinical indications, 18.1% prior CAD	TTE, median time was 3 days	50.8–70.3 72.2% male	♢ LV Function ♢ Segmental LV Wall Motion ♢ RV size and systolic function	♢ 34.7% LVEF ≤ 50% ♢ 40.3% RV systolic function ↓ ♢ RV systolic dysfunction was more common than LV systolic dysfunction ♢ patients with elevated hs-cTnT and elevated NT-proBNP were more likely to exhibit reduced LV function
Dweck et al., June 2, 2020 (19)	Prospective international survey	69 countries	April 3 to 20, 2020	1,216, of whom 813 had confirmed COVID-19, and 298 had a high probability when scanning, 26% prior cardiac disease	TTE	52–71 70% male	Ventricular sizes and function	♢ 55% abnormal echocardiogram ♢ 39% LV abnormalities ♢ 33% RV abnormalities ♢ 3% new myocardial infarction ♢ 3% myocarditis ♢ 2% takotsubo cardiomyopathy 15% severe cardiac disease (severe ventricular dysfunction or tamponade)
Rath et al., May 28, 2020 (20)	Prospective study	University Hospital of Tübingen, Germany	February to March, 2020	123 hospitalized patients (Non-survivors 16 and survivors 107), 22.8% prior CAD	TTE, examed in 24 h	68 ± 15 70% male	♢ LVEF ♢ RV function (TAPSE, RV-FAC) ♢ Aortic stenosis/regurgitation ♢ Mitral regurgitation ♢ Tricuspid regurgitation	♢ Mean LV function 57% ♢ 48.9% RV dilatation ♢ 30.6% tricuspid regurgitation > 1 ♢ RV-FAC ↓ in non-survivors ♢ Visually estimated impaired RV function ↑ in non-survivors ♢ Impaired LV and RV function, and tricuspid regurgitation > grade 1 were significantly associated with higher mortality
Pagnesi et al., July 1, 2020 (21)	Single-center, observational, cross-sectional study	San Raffaele Scientific Institute in Milan, Italy	March 24, 2020 to April 29, 2020	200 non-ICU inpatients, 7.5% prior CAD, 8.5% prior MI	TTE	55–74 65.5 male	♢ RVEDD ♢ RV length ♢ TAPSE ♢ S'TDI ♢ SPAP ♢ Tricuspid regurgitation	♢ 12% PH, 14.5% RVD ♢ PH (and not RVD) was associated with signs of more severe COVID-19 and with worse in-hospital clinical outcome
D' Andrea et al., June 17, 2020 (22)	Prospective study	4 centers in Italy: “Umberto I Hospital, Monaldi Hospital, M. Scarlato COVID Hospital, Cardarelli Hospital	February 20, 2020 to April 20, 2020	115, 26 of whom suffering cardiac injury	TTE	20–88 60% male	♢ RV tract diameter ♢ Tricuspid Peak E/A ratio ♢ TRV ♢ PASP ♢ MPAP ♢ TAPSE	♢ RV function and pulmonary pressures as independent predictors of COVID pneumonia mortality ♢ Patients with PH and RVD had more frequently a history of prior cardiac comorbidities ♢ Only patients with PH showed signs of more severe SARS- CoV-2 infection
Vasudev et al., July 26, 2020 (23)	Retrospective study	Three hospitals in Northern New Jersey, USA	March 15, 2020 to April 15, 2020	45 hospitalized patients, 20% prior ACS	TTE, during hospitalization	61.4 ± 12.2 51% male	♢ Ventricular size and function ♢ SPAP ♢ Pressure and volume overload	♢ 31.1% LVEF ↓ ♢ 11.1% RVEF ↓ ♢ 13.3% RV dilatation ♢ 22.2% PH ♢ Echocardiography is essential for assessment of COVID-19
Baycan et al., August 8, 2020 (24)	Prospective, single-center study	Goztepe Training and Research Hospital, Istanbul, Turkey	April 15, 2020 to April 30, 2020	100 hospitalized patients, all of whom having normal LVEF (≥50%)	TTE, examed on the first day	55.6 ± 14.4 50% male	♢ LV-GLS ♢ RV-FAC ♢ RV-LS ♢ TAPSE ♢ SPAP	♢ LV-GLS and RV-LS were lower in the severe group compared to the non-severe group ♢ LV-GLS and RV-LS are independent predictors of in-hospital mortality in patients with COVID-19 ♢ RVD is important in determining circulation and respiratory management strategies
Krishnamoorthy et al., August 4, 2020 (25)	Single-center study	The Zena & Michael A Wiener Cardiovascular Institute, New York, USA	–	12, 5 of whom required intubation and/or died, 16.7% prior CAD	TTE	29–60 41.7% male	♢ LVGLS ♢ RVGS ♢ RVFWS ♢ RVSP	♢ 41.7% RVD ♢ 58.3% LVD ♢ RVGS and RVFWS were significantly decreased in the patients who had poor outcomes compared with those who did not ♢ LVGLS was decreased regardless of outcome
Van den Heuvel et al., July 8, 2020 (26)	Single center, cross-sectional study	Radboud University Medical Center, Nijmegen, The Netherlands	April 1, 2020 to May 12, 2020	51 hospitalized patients (ICU 19 and non-ICU 32), 22% prior Cardiac history	TTE	51–68 80% male	♢ LV and RV dimensions ♢ LV function (LVEF, GLS) ♢ RV function (TAPSE, RV S') ♢ Atrial dimensions	♢ 27% LVD ♢ 10% RVD ♢ No relation between elevated Troponin T or NT-proBNP and ventricular dysfunction ♢ Ventricular dysfunction by means of L VEF, GLS, TAPSE and RV S' were not significantly different between ICU and non-ICU patients
Zeng et al., July 28, 2020 (27)	Single-center retrospective study	Shenzhen Third People's Hospital, China	January 11, 2020 to April 1, 2020	416 (ICU 35 and non-ICU 381), 3% prior CAD	TTE, only for severe patients (ICU 31 and non-ICU 26)	33–68 47.6% male	♢ LV and RV sizes ♢ LV and RV function ♢ PASP ♢ Ventricular wall thickness	♢ Ventricular wall thickening ♢ LVEF ↓ in 5 (16%) ICU patients ♢ PASP ↑ in 9 (29%) ICU patients ♢ RV dilatation and RVD in 3 (10%) ICU patients

*ARDS, acute respiratory distress syndrome; CAD, coronary atherosclerotic heart disease; COVID-19, coronavirus disease 2019; CVD, cardiovascular disease; ICU, intensive care unit; IHD, ischemic heart disease; LV, left ventricular; LVD, left ventricular dysfunction; LVEF, left ventricular ejection fraction; LVGLS, left ventricular global longitudinal strain; MPAP, mean pulmonary artery pressure; PASP, pulmonary artery systolic pressure; PH, pulmonary hypertension; RV, right ventricular; RVD, right ventricular dysfunction; RVEDD, right ventricular end-diastolic diameter; RVEF, right ventricular ejection fraction; RVFAC, right ventricular fractional area change; RVFWS, right ventricular free wall strain; RVGS, right ventricular global strain; RVLS, right ventricular longitudinal strain; RV S', right ventricular systolic excursion velocity; RVSP, right ventricular systolic pressure; SPAP, systolic pulmonary artery pressure; S' TDI, tissue Doppler imaging S wave; TAPSE, tricuspid annular plane systolic excursion; TRV, tricuspid regurgitation velocity; TTE, transthoracic echocardiography*.

### MRI Findings

MRI can be used to quantitatively assess myocardial fibrosis and oedema (28, 29). This technique is currently the gold standard for evaluating cardiac morphology and function (30). MRI analysis includes conventional sequences and quantitative mapping sequences. Conventional sequences include short-axis and long-axis cine, T2-weighted imaging (T2WI), and late gadolinium-enhanced scanning (LGE). Quantitative mapping sequences include native T1/T2 mapping and post-contrast T1 mapping. T1 mapping is mainly applied to quantitatively assess diffuse fibrosis, while T2 mapping enables the quantification of edema. Post-contrast T1 mapping can better obtain extracellular volume fraction, which can be used as the most sensitive biomarker of myocardial fibrosis and is highly consistent with histopathological findings (31). Myocardial oedema is assessed on T2WI images, and LV and RV functional parameters are calculated by changes in endocardial and epicardial contours (14). A study of competitive athletes recovered from COVID-19 found that cardiac MRI (CMR) was more sensitive to identify myocarditis, helping to identify the high-risk population. CMR has a negative predictive value for exclusion of myocarditis (16). Two other studies, analyzing of patients who had already recovered from COVID-19 when undergoing MRI, showed increased T1 and T2 signals, positive LGE and/or pericardial enhancement in 58–78% of the population (14, 15). In Huang's study, 26 patients without previous cardiac diseases were all recovered and isolated for 14 days, and myocardial edema was found in 54% of patients (14). In Puntmann's study, mostly non-hospitalized patients recovered from COVID-19, 60% of them found myocardial inflammation (16). While COVID-19 patients had cardiac injury, regardless of preexisting disease, severity and overall course of COVID-19 manifestations, time since initial diagnosis, or presence of cardiac symptoms (16). Decreased RV functional parameters, including the RV ejection fraction, cardiac output, the cardiac index, and stroke volume, were found in patients with positive cardiac MRI findings compared with healthy controls (*P* < 0.05). These findings suggest that sustained cardiac involvement, including oedema, fibrosis, and impaired RV contractile function, may remain in patients who recover from COVID-19. Similarly, Puntmann et al. showed that the RV ejection fraction was decreased in patients with COVID-19 compared with healthy controls (15). They also found a reduction in the LV ejection fraction in the recovered COVID-19 cohort. However, Huang et al. showed that LV function was hyperdynamic or normal in the same subgroup (14). The outcomes were inconsistent between these two studies. Regardless of the discrepancy, Puntmann et al. considered that native T1 and T2 were the best indicators with the ability to detect COVID-19-related myocardial pathology (15). Further investigation on the long-term cardiovascular consequences of COVID-19 is required (16).

### Echocardiographic Findings

Echocardiography is commonly used for assessing cardiac damage. This technique is easier to perform than cardiac MRI. Conventional echocardiographic evaluation includes cardiac structural assessment, myocardial systolic and diastolic function, and valvular hemodynamics. According to the American Society of Echocardiography, RV dysfunction is present when the following parameters used to quantify RV function are less than low values in the normal range: pulsed Doppler systolic myocardial velocity <9.5 cm/s, tricuspid annular plane systolic excursion <17 mm, RV ejection fraction <45%, and RV fractional area change <35% (32, 33). RV dilatation is usually observed early in the pressure-overloaded right ventricle. Typically, in the RV-focused view, a basal diameter > 41 mm and an intermediate horizontal diameter > 35 mm indicate RV dilatation (32).

Most inpatients with COVID-19 have RV dilatation or dysfunction. However, LV dysfunction is less common. In a study of 74 patients with COVID-19, 27% presented with RV dysfunction, but LV function was hyperdynamic or normal in 89% (6). Szekely et al. (5) showed that RV dysfunction was more common in patients with elevated troponin levels and a poor clinical grade, whereas the total number of patients with an impaired LV function was relatively smaller. Notably, in several other studies, LV dysfunction was not rare in patients with COVID-19 (18, 23, 25). This discrepancy among studies may be due to differences in the study populations, but RV damage is still universally found by echocardiography in patients with COVID-19. We summarized the results of recent cardiac imaging studies (Table 2). Among patients with COVID-19-related myocardial injury, the proportion of RV dilatation ranged from 13.3 to 48.9% (5, 6, 17, 20, 23). RV dilatation associated with elevated D-dimer levels and C-reactive protein levels was more common in patients with COVID-19 (6, 17, 18, 20). There was no significant difference in the incidence of major comorbidities (hypertension, diabetes and known coronary artery disease), laboratory markers of inflammation (white blood cell count, C-reactive protein) or myocardial injury (troponin) in patients with right ventricular dilatation (17).

Conventional echocardiographic parameters are not sensitive to early RV systolic dysfunction, and therefore, cannot be used for early diagnosis (34). Two-dimensional speckle tracking echocardiography can more accurately evaluate myocardial function and detect subclinical cardiac functional impairment earlier than conventional echocardiography (35, 36), which can measure LV global longitudinal strain (LVGLS), RV longitudinal strain (RVLS), RV free wall strain (RVFWS), and RV global strain (RVGS). In a retrospective study, RVLS was found to predict mortality in patients with COVID-19 more accurately. Therefore, there is potential value of RVLS for risk stratification in COVID-19. The optimal cut-off values for prediction of outcome were calculated to be −23% for RVLS, 43.5% for RV fractional area change, and 23 mm for tricuspid annular systolic displacement (7). Baycan et al. (24) and Krishnamoorthy et al. (25) also evaluated the prognostic value of strain indices. RVGS and RVFWS were significantly reduced in patients with poor clinical outcomes. RVLS is an independent predictor of in-hospital mortality in patients with COVID, while the predictive value of LVGLS for mortality varies in different studies. However, speckle-tracking echocardiography is demanding on image quality. The structure of the chest wall in different patients has a large effect on imaging, and critically ill patients are unable to cooperate in adjusting positions, both of which affect the results.

### RV Dysfunction and Prognosis in COVID-19

Cardiac imaging findings have shown that RV damage is common in patients with COVID-19. Concomitant RV damage usually indicates a poor prognosis and affects the clinical outcome of patients. In a study of 120 COVID-19 cases, non-survivors showed elevated pulmonary artery systolic pressure, dilated right heart chambers, and diminished RV function compared with survivors (7). In another study where 28 patients died of COVID-19, 14 had a RV abnormality, but only 2 had LV impairment (6). Indeed, these outcomes all indicate a strong relation between RV dysfunction and poor prognosis. One multivariate analysis revealed that RV enlargement was the only factor significantly associated with mortality (17). Patients with COVID-19 and RV dysfunction often have more severe symptoms (19). Argulian et al. found that renal dysfunction was more common in patients with RV dilatation than those without (17). Therefore, RV dysfunction often predicts the presence of some severe complications, and they may partly account for the high mortality in this population. Additionally, Pagnesi et al. (21) showed that pulmonary hypertension, instead of RV dysfunction, was associated with worse in-hospital clinical outcomes in patients with COVID-19. However, because their study population was non-intensive care unit patients without mechanical ventilation, this may have eliminated the association between COVID-19 and RV involvement.

Although CMR imaging is the gold standard for assessing RV function (30), the high infectivity of COVID-19 and the inability of patients to hold their breath for a long time limit its application. Patients without pre-existing cardiovascular diseases are more likely to have normal echocardiography than those with pre-existing cardiovascular diseases (21). RV dysfunction is more common than LV dysfunction in COVID-19 (23). Patients with RV dysfunction had a higher rate of cardiac comorbidities compared with patients without RV dysfunction (37). The main reasons for performing echocardiography in the previous study were suspected heart failure and elevated cardiac biomarker concentrations (5, 21, 23). Independent predictors of RV abnormalities are suspected RV failure and moderate or severe COVID-19 symptoms (21). To minimize the risk of the spread of infection, at least echocardiography should be performed in patients with suspected heart failure, more cardiac comorbidities, elevated cardiac biomarkers, and severe COVID-19 symptoms. Abnormal transthoracic echocardiography ultimately affects decision-making of clinicians in 16–33.3% of patients (18, 19). It also showed that clinical management was altered in 24.2% of patients because of acute cardiovascular events observed with transthoracic echocardiography (18).

Male was an independent predictor of prognosis (7), while age, weight, and ethnicity were not significantly different in COVID-19 patients with cardiac injury. Patients with a history of established cardiovascular disease or elevated cardiac biomarkers have an increased susceptibility to infection and an increased risk of severe disease progression and death (4, 37, 38). These patients are more likely to have RV dysfunction and pulmonary hypertension, which are independent risk factors for poor prognosis (21, 22). The proportions of echocardiographic abnormalities and serious heart disease are similar after excluding patients with pre-existing heart disease (heart failure, valvular disease, or ischemic heart disease), suggesting that cardiac abnormalities are associated with COVID-19 infection in this population (19).

## Aetiology of COVID-19 With RV Functional Changes May Involve Multiple Facets

### The Right Ventricle Is More Susceptible to Lung Injury Than the Left Ventricle

The transverse section of the right ventricle is crescent-shaped compared with the thick wall of the left ventricle, and the relative surface area of the right ventricle is higher and the volume is lower. The thin RV free wall has greater compliance than the left ventricle. These anatomical features allow acute dilatation of the right ventricle when there is a sharp increase in afterload. RV systolic function is sensitive to increased pressure, and a slight rise in pulmonary circulation resistance causes RV overload and impaired systolic function. The primary target organ of severe acute respiratory syndrome coronavirus-2 is the lungs. The right ventricle is vulnerable to a slight increase in pulmonary vascular resistance (39), making it more vulnerable to injury than the left ventricle. As the right ventricle continues to expand, RV geometry changes, and the tricuspid annulus dilates insufficiently, resulting in tricuspid regurgitation. Tricuspid regurgitation leads to further RV dilatation and volume overload, which shifts the interventricular septum to the left and affects LV filling and contraction. RV pressure overload increases wall tension, increases myocardial oxygen consumption, and decreases RV oxygen supply during systole. This further leads to myocardial ischaemia and reduces RV contractility. RV dilatation may precede development of acute cor pulmonale (40).

### Acute Respiratory Distress Syndrome and RV Dysfunction

COVID-19 mainly affects the respiratory system and the incidence of acute respiratory distress syndrome (ARDS) reported in COVID-19 ranges from 19.6 to 31% (37, 38, 41). ARDS is a severe form of COVID-19, which leads to a dramatic increase in RV afterload and delayed contraction owing to its own pathological effects and mechanical ventilation with a high positive end-expiratory pressure (PEEP). This then reverses the end-systolic transseptal pressure gradient. The incidence of RV dysfunction in ARDS has been reported to be 22–50% (33). There is no robust evidence to verify a definitive causal relationship between RV dysfunction and mortality in ARDS. However, RV dysfunction is undoubtedly associated with increased mortality and poorer prognosis in patients with COVID-19-related ARDS (42). In the setting of ARDS, numerous factors can destroy the pulmonary circulation, including mechanical compression by interstitial oedema, microvascular thrombosis, hypoxic or mediator-induced pulmonary vasoconstriction, and pulmonary vascular muscular remodeling. These factors raise pulmonary arterial pressure and further rapidly increase RV afterload. Pulmonary vascular resistance abates RV ejection and LV pulmonary venous return, while RV dilatation results in LV compression by a septal shift because of an inextensible pericardium. Both of these mechanisms account for the decrease in LV ejection and RV coronary blood flow. Therefore, ARDS-derived pulmonary circulation injury in COVID-19 has a deleterious effect on RV dysfunction (43, 44).

RV dilatation secondary to mechanical ventilation during hospitalization for ARDS requires attention. In the ARDS population, a lung protective ventilation strategy is recommended and mainly refers to PEEP. High PEEP levels cause overinflation of the normal alveoli and compression of intra-alveolar vessels, which lead to high pulmonary vascular resistance and increased RV afterload (43). Therefore, RV dysfunction can be a haemodynamically significant and deleterious consequence of COVID-19-related mechanical ventilation. Notably, Sud et al. showed that there was no meaningful correlation between PEEP and RV dilation on echocardiography in their COVID-19 infection cohort (17). However, they did not deny the possible contribution on RV dilatation from mechanical ventilation.

### Pulmonary Embolism and RV Dysfunction

Owing to risk factors, such as virus-induced endothelial injury, vascular inflammation, and hospitalization-related prolonged immobilization, most patients with COVID-19 stay in a hypercoagulable state, and they are vulnerable to venous thrombosis. Poissy et al. studied 107 patients with COVID-19 who were admitted to the intensive care unit (45). They reported a high incidence of pulmonary embolism (20.4%), which was significantly higher than the contemporaneous average level in patients with influenza and in in-hospital patients. An autopsy of patients with COVID-19 showed a high incidence of deep venous thrombosis (58%) and death-causing pulmonary embolism (33%) (46). When thrombus enters pulmonary vessels, it produces mechanical obstruction and stimulates endothelial cells and platelets to release vasoactive mediators (e.g., thromboxane A2, serotonin). This triggers obstruction-related vasoconstriction and increases RV afterload and pulmonary arterial pressure in patients. Oxygen demand from the right ventricle increases, while embolism-associated hypoxemia and hypotension decrease myocardial oxygen supply. This imbalance finally leads to RV dysfunction (47).

### Myocardial Injury and a Cytokine Storm

Myocardial injury was recognized early in patients with COVID-19 in China, and it also partly accounts for RV dysfunction. Myocarditis can occur before pulmonary symptoms of shock (48). The possible mechanisms for myocardial injury are as follows. Angiotensin-converting enzyme 2 (ACE2) is highly expressed not only in the lungs, but also in the cardiovascular system, thus possibly mediating viral entry into cardiomyocytes to cause direct damage. Cardiac elevation of troponin-I levels is accompanied by an increase in other inflammatory markers, such as lactate dehydrogenase, ferritin, tumor necrosis factor-α (TNF-α), interleukin-6 (IL-6) and interleukin-8 (IL-8). This could represent a cytokine storm syndrome or secondary haemophagocytic lymphohistiocytosis, which may result in cardiac involvement (49). After viral invasion into the body, T cells become activated, and they produce and release amounts of antiviral cytokines. Because of an imbalanced response among subtypes of T helper cells, a cytokine storm release is induced, which attributes to hyperactivation of monocytes/macrophages. This then leads to tissue damage to multiple organs and causes complications, such as ARDS and cardiac insufficiency.

In ARDS, increased levels of cytokines, such as IL-6, IL-8, TNF-α, can be tested. In particular, IL-6 is an important marker. A previous study reported that elevated circulating IL-6 levels were associated with increased mortality in COVID-19 (50). Targeted therapy against the IL-6 receptor with tocilizumab can be effective in severe COVID-19 cases. A cytokine storm is essentially a protective response to limit spread of the virus, but its exact mechanism of myocardial injury remains unclear. However, cardiomyocyte and endothelial cell death triggered by inflammatory cytokines, such as TNF-α, has been well-documented (51). Ventricular dilatation with a reduction in the ejection fraction may be an adaptive response to myocardial dysfunction. Myocardial depression results from the direct or indirect action of one or more cardioinhibitory substances. Besides, TNF-α and IL-1, which act as potent inducible nitric oxide synthase inducers, are associated with inhibition of cardiomyocyte function. For one thing, nitric oxide interferes with calcium metabolism in cardiomyocytes, which in turn impairs contractile function. For another, peroxynitrite generated by interaction of nitric oxide with superoxide ions is directly toxic to cardiomyocytes (52). Additionally, Hypoxemia caused by COVID-19 can also induce intracellular calcium overload, leading to apoptosis of cardiomyocytes (53). So, an inflammatory storm, as well as autoimmune activation, can induce extensive vascular and myocardial inflammation, while predisposing to diffuse thrombosis (54).

**Figure 1:**
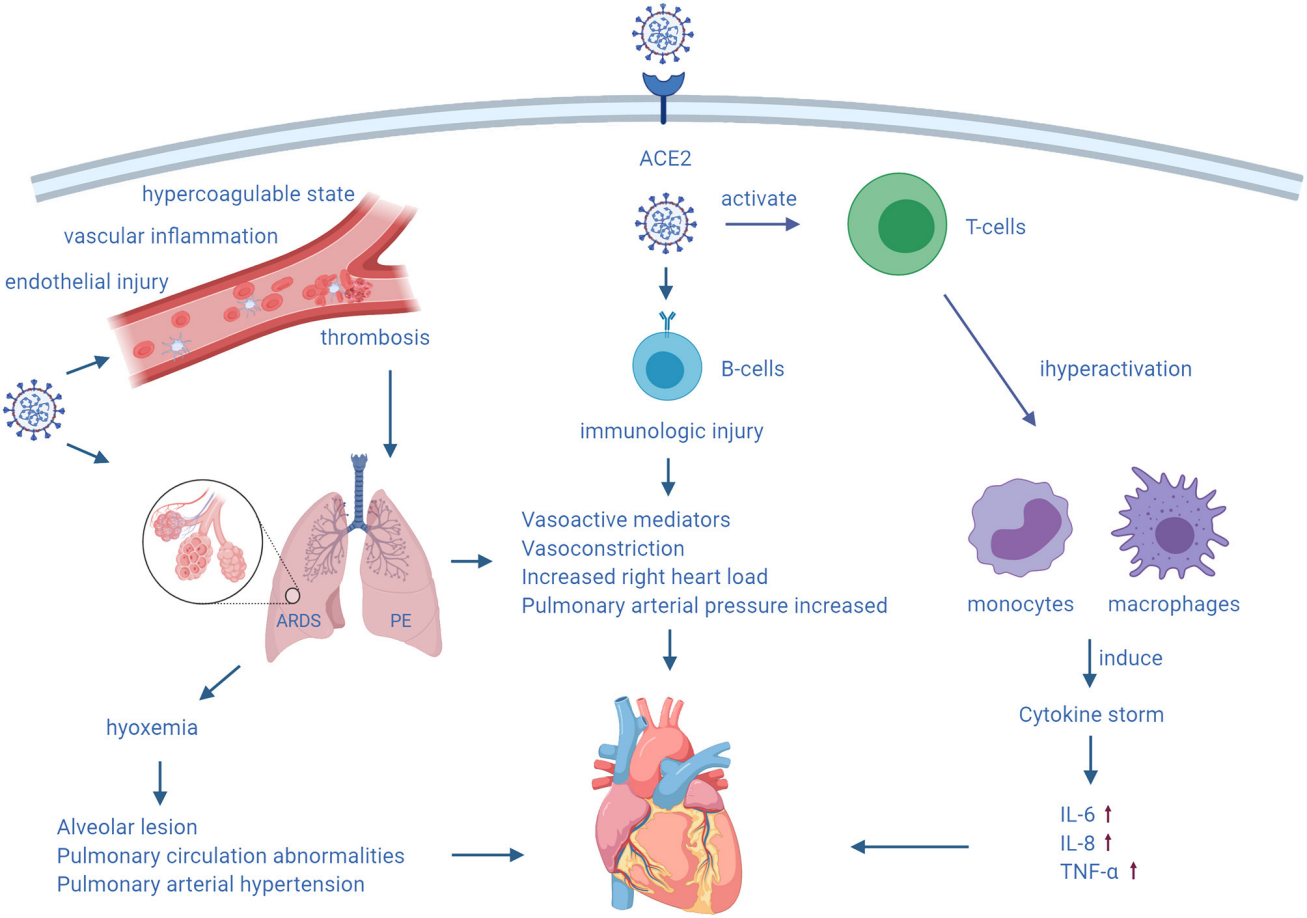
Mechanism of RV damage caused by COVID-19.

In summary, the mechanism of myocardial injury varies at different stages of COVID-19. Isolated RV dysfunction can be found in the presence of severe ARDS or pulmonary embolism (55), while diffuse myocardial damage caused by viral toxicity and the host immune response also partly weaken RV function (Figure 1). Because of ACE2 expression in the endothelium, virus-induced endothelial shedding and microvascular damage may lead to thrombosis and myocardial infarction (55). ACE2-mediated direct injury may be a major mechanism in the early stages of COVID-19. With aggravation of COVID-19, pulmonary and cardiac injury caused by hypoxia is gradually aggravated. Inflammatory reactions and autoimmune damage leading to exacerbation of disease play a major role in the later stages of COVID-19.

## Treatment of RV Dysfunction With COVID-19

### Medical Treatment

Medical treatment of RV functional impairment includes reducing volume load, enhancing RV contractility, and reducing pulmonary arterial pressure. Diuretics can reduce intravascular volume. The RV Starling curve is flat, and improvement in RV function can only be observed with a large negative fluid balance. Normally, the RV filling pressure needs to be maintained at a slightly increased level at ~8–12 mmHg. The volume status can be further adjusted on this basis to achieve optimal RV function and cardiac output (56). RV pressure monitoring is also important when circulating hypovolemia results in decreased blood pressure and the requirement for appropriate fluid replacement. Central venous pressure and mixed venous oxygen saturation help determine RV filling and oxygen supply. Echocardiography also helps determine the volume status. RV dilatation with restriction of LV filling indicate excessive preload.

Levosimendan is a novel calcium sensitizer that stabilizes the spatial configuration of myocardial fibrin and increases myocardial contractility. This calcium sensitizer has the advantages of no effect on diastolic function or arrhythmia, and does not increase myocardial oxygen consumption. Levosimendan improves RV myocardial contractility and reduces RV afterload. Morelli et al. showed that levosimendan was an effective treatment option for ARDS with acute right heart dilatation, and it was believed to dilate the pulmonary circulation and improve RV contractility (57). Norepinephrine might improve RV function by restoring RV perfusion pressure as suggested in an experimental model of massive pulmonary embolism (58). Intravenous epoprostenol can improve symptoms, hemodynamics, and the survival rate, and enhance RV systolic function (59). Bosentan is a specific endothelin receptor antagonist, which reduces mean pulmonary arterial pressure and increases the cardiac index. Inhaled nitric oxide can selectively dilate pulmonary vessels, improve the ventilation-blood flow ratio, significantly reduce pulmonary vascular resistance, and increase cardiac output, while it has a slight effect on systemic vascular resistance. In patients with pulmonary heart disease caused by ARDS, inhaled nitric oxide reduces pulmonary arterial pressure and pulmonary inflammatory responses (60). In patients with pulmonary embolism and ARDS, prostacyclin is as effective as inhaled nitric oxide in reducing pulmonary arterial pressure, improving gas exchange and oxygenation, increasing cardiac output, and improving RV function (61, 62).

To alleviate inflammation and fibrosis, corticosteroids are considered as potential therapeutic agents for ARDS, which reduce morbidity and mortality, but remain controversial. High-dose corticosteroid therapy can accelerate improvement of ARDS, reduce mortality, and shorten the duration of invasive mechanical ventilation (63). However, the World Health Organization recommends that systemic corticosteroids should not be routinely used in patients with COVID-19 or COVID-19-associated ARDS (64).

Severe COVID-19 is often associated with thrombosis, and disseminated intravascular coagulation may be present in the majority of fatal cases (65). Prolonged immobilization and hormonal therapy increases the risk of venous thromboembolism. Patients with right heart enlargement are also prone to cardiac thrombosis. Coagulopathy due to COVID-19 may be associated with bacterially-induced infectious coagulopathy. Overproduction of inflammatory cytokines, vascular endothelial injury, and increased levels of damage-associated molecular patterns contribute to thrombosis. Patients who meet the sepsis-induced coagulopathy score criteria or have significantly elevated D-dimer levels may benefit from anticoagulant therapy by mainly using low-molecular-weight heparin (66). Among 449 patients with severe COVID-19, 99 received heparin (mainly low-molecular-weight heparin) for 7 days or longer, and 28-day mortality was significantly lower in patients with sepsis-induced coagulopathy scores ≥ 4 or D-dimer levels > six times the upper limit of normal using heparin than in non-users (*P* = 0.029, *P* = 0.017). Lin et al. (67) also recommended the use of low-molecular-weight heparin in patients with D-dimer values > four times the upper limit of normal. Thrombotic coagulopathy is common in severe patients with COVID-19, and D-dimer is more useful than other coagulation markers for prediction of this disease. However, bleeding complications are relatively uncommon in COVID-19. Therefore, anticoagulant therapy is necessary.

### Device-Assisted Therapy

Mechanical ventilation, sedation, and analgesia may lead to increased afterload, increased transpulmonary pressure, and decreased cardiac output. Therefore, mechanical ventilation indications need to be strictly followed. For critically ill patients requiring mechanical ventilation, appropriate mechanical ventilation measures should be implemented to avoid hypoxemia, hypercapnia, a low or high lung volume, and high PEEP. Protective ventilation strategies should also be used when necessary. The principle of mechanical ventilation in patients with right heart failure is to limit plateau pressure and offer PEEP, avoiding hypercapnia, hypoxemia, and pulmonary vasoconstriction. Respiratory settings are adjusted according to the tolerance of the right ventricle, as assessed by ultrasound, to coordinate the balance between recruitment and hyperventilation resulting from ventilation according to RV function (68). PEEP can dilate the alveoli, compress extra-alveolar capillaries, and cause an increase in pulmonary vascular resistance. This increases afterload and RV volume, resulting in RV dilatation, which in turn affects LV filling. Appropriate PEEP is important for treatment.

**Figure 2:**
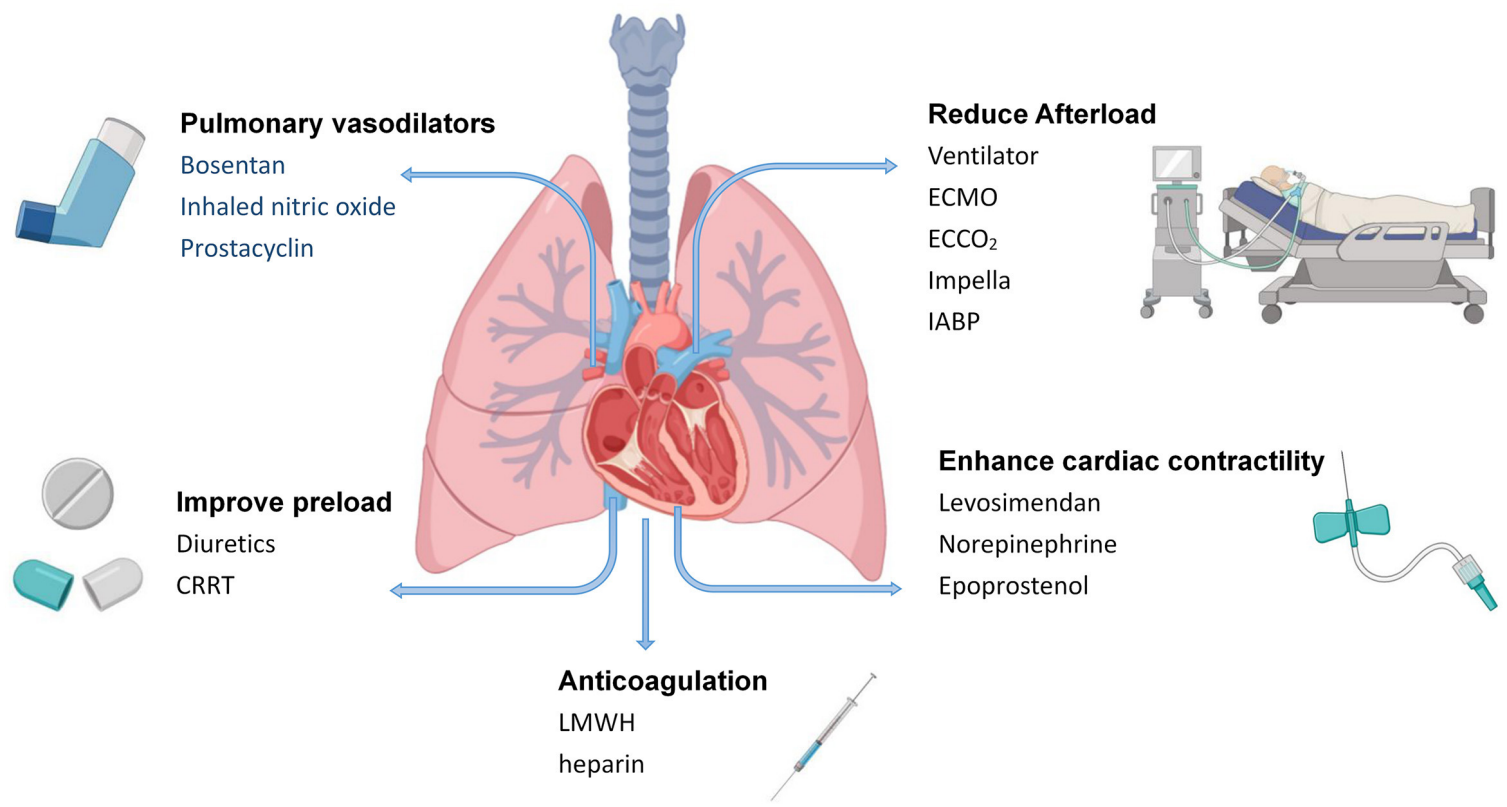
Treatment of RV dysfunction with COVID-19. ECCO_2_, extracorporeal carbon dioxide removal devices; IABP, intra-aortic balloon pump; LMWH, low molecular weight heparin.

When optimized ventilation measures still do not improve hypoxemia in mechanically ventilated patients, extracorporeal membrane oxygenation (ECMO) can be considered. ECMO is used as a rescue therapy for COVID-19 with refractory hypoxemia in accordance with provisional guidelines established by the World Health Organization (69) in 2020. However, because of a lack of relevant trials on the use of ECMO in patients with COVID-19, there is insufficient evidence that these patients can benefit from ECMO. For acute RV failure caused by severe ARDS, extracorporeal carbon dioxide removal devices can be considered for super-protective lung ventilation (tidal volume: 4 mL/kg) (70). Additionally, increased work of breathing, pulmonary oedema, and endogenous PEEP caused by weaning increase RV afterload. Worsening of RV function is an important cause of weaning failure in mechanically ventilated patients. When RV function is impaired in combination with severely impaired LV function, adjunctive therapy with Impella device or intra-aortic balloon counterpulsation can be used. Continuous renal replacement therapy may be considered when volume overload and drug therapy are not effective. COVID-19-related myocardial injury treatments are summarized in Figure 2.

## Future Directions and Conclusions

RV dysfunction usually indicates a poor prognosis in the wide array of cardiopulmonary diseases. Assessment of RV function is essential for managing ARDS, acute pulmonary embolism, and pulmonary hypertension. RV dilatation is common in patients with COVID-19. A full understanding of COVID-19-related RV dysfunction is conducive for early identification and precise treatment, and to help improve the prognosis of severe cases and reduce mortality. Early recognition of RV dysfunction allows appropriate treatment to be provided as soon as possible. How to identify RV dysfunction early is important for stratification of disease risk and prognostic evaluation. Echocardiography, cardiac MRI, right heart catheterization, and other examinations are helpful for early identification of RV dysfunction. At the same time, monitoring of biological indicators related to RV function, such as troponins and brain natriuretic peptide, should not be ignored for the suggestive role in RV function. It is recommended to assess RV function as soon as possible, for COVID-19 patients with suspected cardiac injury, elevated cardiac biomarkers, severe respiratory symptoms. RV function is often monitored to optimize haemodynamic and respiratory parameter settings. Timely medical treatment should be delivered. And device assistance should be implemented if necessary. RV damage reflects an association between myocardial injury and COVID-19. In future medical care, clinicians need to further focus on the morbidity of RV dysfunction in patients with COVID-19. Using cardiac imaging to detect RV dysfunction will provide early information concerning the severity of COVID-19 infection. Performing an appropriate strategy of the right ventricle will be helpful to reduce mortality and improve prognosis in this persistent epidemic.

## Author Contributions

WL contributed conception and constructing the overall structure and contents. YL wrote the draft and sections of the manuscript. All authors contributed to manuscript revision, read, and approved the submitted version.

## Conflict of Interest

The authors declare that the research was conducted in the absence of any commercial or financial relationships that could be construed as a potential conflict of interest.

## Abbreviations

ARDS: acute respiratory distress syndrome
COVID-19: coronavirus disease 2019
ECMO: extracorporeal membrane oxygenation
LV: left ventricular
LVEF: left ventricular ejection fraction
LGE: late gadolinium enhancement
MRI: magnetic resonance imaging
PEEP: positive end-expiratory pressure
RV: right ventricular
RVLS: right ventricular longitudinal strain.
